# Comparison of Reported Fatalities, Falls and Injuries in Thoroughbred Horse Jumps and Flat Races in the 2022 and 2023 Jumps Race Seasons in Victoria, Australia

**DOI:** 10.3390/ani14050804

**Published:** 2024-03-05

**Authors:** Angela Jeppesen, Rebekah Eyers, Di Evans, Michael P. Ward, Anne Quain

**Affiliations:** 1 School of Veterinary Science, University of Sydney, Camperdown, NSW 2006, Australia; 2 RSPCA South Australia, Stepney, SA 5069, Australia; 3 RSPCA Australia, Deakin, ACT 2600, Australia

**Keywords:** jumps racing, hurdle, steeplechase, Thoroughbred, animal welfare

## Abstract

**Simple Summary:**

Jumps racing is a form of Thoroughbred horse racing that involves hurdles and steeples and typically longer distances and heavier weights compared with flat racing, which does not incorporate obstacles. The continuation of jumps racing remains contentious due to the higher risk of fatalities, falls and injuries for horses, compared with flat racing. In Australia, jumps racing is carried out only in the state of Victoria after a legislated ban on jumps racing in South Australia in 2022. Jumps races account for only 1.8% of Thoroughbred horse races in Victoria. This study compared the incidence of fatalities, falls and injuries in horses participating in hurdle and steeplechase races with those participating in flat races at the same race meets, for all jumps races in the 2022 and 2023 Thoroughbred horse jumps racing seasons in Victoria, Australia. Overall, horse fatalities, falls and injuries occurred at higher rates in jumps races compared with flat races during the study period.

**Abstract:**

Jumps racing is a form of Thoroughbred horse racing that involves hurdles and steeples and typically longer distances, and heavier weights compared with flat racing, which does not incorporate obstacles. In Australia, jumps racing is carried out only in Victoria, one of eight states and territories. The continuation of jumps racing is contentious due to the higher risk of fatalities, falls and injuries for horses, compared with flat racing. While measures have been introduced by the industry to improve the safety of riders and horses, the rates of fatalities, falls and injuries in horses participating in jumps races have not been collectively reported in Australia since the 2012 to 2014 race seasons. Although information on individual horse fatalities, falls and injuries is published by Racing Victoria in Stewards’ Reports, the data are not aggregated, and so cannot readily be used to assess trends or evaluate the efficacy of safety measures introduced by the industry. The aim of this study was to determine the fatality, fall and injury rates for horses participating in hurdle and steeplechase races in Victoria in the 2022 and 2023 Thoroughbred horse jumps racing seasons compared with horses participating in flat races at the same race meets. Data on horse fatalities, falls and injuries were extracted from the published Racing Victoria race results and Stewards’ Reports for the jumps races (*n* = 150) and corresponding flat races (*n* = 157) held at the 38 jumps race meets in Victoria in 2022 and 2023. Overall, horse fatalities, falls and injuries occurred at higher rates in jumps races compared with flat races during the study period. The rate of horse fatalities in jumps races was 3.3 per 1000 starts, with no fatalities in flat races. The rate of horse falls in hurdle races was 24 per 1000 starts and 41.6 per 1000 starts in steeplechase races, comparable with rates previously reported in the 2012 to 2014 seasons. There were no falls in flat races. Horse injuries occurred at a rate of 68.9 per 1000 starts in jumps races compared with 18.8 per 1000 starts in flat races. In hurdle and steeplechase races, veterinary clearance being required following horse injury was 5.4 times (OR 5.4, 95% CI 2.8–10.2) and 7.2 times (OR 7.2, 95% CI 3.3–15.6) more likely, respectively, compared with flat races. The risk of trauma was 4 times more likely in hurdle and steeplechase races (OR 4.8, 95% CI 1.7–13.3 and OR 4.1, 95% CI 1.2–13.4, respectively) and the risk of lameness was increased by 2.5 times in hurdles (OR 2.5, 95% CI 1.2–5.2) and 5.1 times in steeplechase races (OR 5.1, 95% CI 2.3–11.5), compared with flat races. These findings support concerns about the welfare of horses involved in jumps racing and of the need for further safety measures to reduce these risks.

## 1. Introduction

Jumps racing is a form of Thoroughbred racing that involves hurdles and steeplechase. In hurdle races in Victoria, horses are required to jump over a series of hurdles that are a maximum of 1 metre high, over distances between 3200 m and 4200 m. Steeplechase races typically incorporate higher obstacles (up to 1.15 m fences) and run for longer distances (between 3200 m and 5500 m) [1,2]. By comparison, most flat races are between 1000 m and 3200 m [3]. Horses in jumps races are also required to carry heavier weights (64–69 kg for jumps jockeys) than in flat races (45–59 kg) [1,4]. The obstacles and longer distances contribute to jumps races being considered inherently higher risk than flat races [5] with consequent implications for horse safety and welfare. Jumps racing continues to be carried out in 16 countries, including Australia [6].

In 2021/2022, jumps races accounted for 0.4% of races in Australia and 1.8% of races in Victoria [7]. Victoria is the only jurisdiction out of eight states and territories in Australia in which jumps racing is carried out after the legislated ban of jumps racing in South Australia in 2022 [8]. In Victoria, the race program typically comprises 19 jumps race meets held annually between March and August [9], predominantly in key rural and regional areas in the southwest of the state [2]. Despite the small scale of jumps racing in Australia and Victoria, Racing Victoria Limited (RV) has committed to continuing jumps racing throughout the next decade [10].

The continuation of jumps racing remains the subject of ongoing debate due to the higher risk of fatalities, falls and injuries for horses, compared with flat races [11,12]. The rates of horse fatalities in jumps races in Australia, the UK and the USA have been reported to be between 3.4 and 14.3 per 1000 starts, in contrast to flat races of between 0.44 and 2.5 per 1000 starts [13]. In Australia, for the 2012 to 2014 racing seasons, the overall fatality rate was 5.1 per 1000 horse starts in jumps races with fatalities in steeplechase races more than 18 times the fatalities in hurdle races [14]. In that study, horse falls were reported at a rate of 29 and 40 per 1000 starts for hurdles and steeplechase races, respectively [14]. In Victoria between 1989 and 2004, catastrophic limb injuries were responsible for 71.6% of horse fatalities in flat and jumps races and 69% of fatalities in jumps races [15].

Track-, horse- and rider-associated risk factors for horse fatalities, falls and injuries in jumps races have been studied in a number of jurisdictions, including Australia, New Zealand, the UK, Canada and the USA [13,14,16,17,18,19,20]. Risk factors studied include the track condition, height and type of the jumps, race speed, race distance, number of runners, age and stage of career, and whip use [13,14,16,17,18,19,20]. Musculoskeletal injury and contact with barriers and other horses have also been identified as key causes of fatalities and falls [21]. Horse falls were reportedly associated with 35–80% of horse fatalities in jumps racing in Australia, the UK and the USA [13].

Historically, there have been a number of reviews into jumps racing in Australia. In 1991, a Commonwealth Senate Select Committee on Animal Welfare report “Aspects of Animal Welfare in the Racing Industry” recommended that jumps racing be phased out [22]. Since then, there have been six further reviews into horse and rider safety in jumps racing between 1994 and 2008 [14] and most recently, a South Australian inquiry in 2016 [5]. Reviews have recommended the introduction of measures aimed at improving horse and rider safety [5,23]. Several of the recommended measures were adopted, including limiting the season to the cooler months of the year (between March and August), limiting the number of horses in a race, removing the final jump of the race, changing the height and angle of jumps, restricting races to track conditions rated better than a Soft 5 or equivalent, and amending the rules to require jockeys to retire horses from the race where the horse is fatigued, not in contention to win, or there is a risk of injury to the horse or rider by attempting to complete the race [1,14]. The Victorian Racing Rules also now require that horses used in jumps racing must be at least three years of age and that horses and riders must undergo training and qualification trials prior to maiden hurdle races. Furthermore, trainers and jockeys must participate in mandatory annual workshops [1].

Measures introduced prior to 2012 were reported to reduce the risk of fatalities and falls in hurdle races in the 2012 to 2014 race seasons, with fatality rates lower than any other consecutive three-year period from 1986 [14]. However, in that study period, the fatality rate in steeplechase races (14 per 1000 starts) remained comparable with or higher than was previously documented [14].

More recently, the risk of horse fatalities in flat races has been reported for other jurisdictions in Australia (New South Wales (NSW) and the Australian Capital Territory (ACT)) [24]. However, a collation of data on the incidence of fatalities and falls in jumps racing in Australia has not been publicly reported since the 2012 to 2014 race seasons [14]. Although Stewards’ Reports contain information on individual horse fatalities, falls and injuries, these data are not aggregated. Nor is there a consistent reporting framework for the classification of injuries. The incidence of injuries and types of injuries, which potentially provide a more comprehensive indicator of outcomes for horses in jumps races, are not currently collated in Australia.

In the context of the recent ban on jumps racing in South Australia, ongoing public debate, lack of publicly available aggregated statistics and ongoing investment in jumps racing in Victoria, it is timely to ascertain the current incidence of horse fatalities, falls and injuries in jumps racing in Victoria, to inform discussions.

The aim of this study was to determine the fatality, fall and injury rates for Thoroughbred horses participating in jumps races compared with flat races in Victoria in the 2022 and 2023 jumps race seasons.

## 2. Materials and Methods

### 2.1. Data Collection

This retrospective study utilized publicly available data obtained from races at the 38 race meets in which jumps races were held in the 2022 and 2023 jumps racing seasons, in Victoria. In 2022 and 2023, all jumps race meets were held between March and August. For each of these race meets, data were extracted for both the jumps races and flat races held at that same race meet. Data were obtained from the Results and Stewards’ Reports area of the Racing.com, last accessed on 9 December 2023, website and the Jumps Review Panel Race Reports (JRP Reports) available on the RV website (https://www.racingvictoria.com.au/the-sport/racing/jumps/jumps-review-panel, last accessed on 9 December 2023).

For each race, data were extracted on the race date, venue (Terang, Warrnambool, Hamilton, Pakenham, Casterton, Lakeside, Sale, Coleraine or Ballarat), type of race (flat, hurdle or steeplechase), number of horses starting the race, number of fatalities, falls, failure to finish, total injuries, slow recovery time, veterinary clearance required, horse contacts, dislodged rider, whip breach, and failure to retire.

A horse fatality was defined as the death of a horse during the race or as a direct result of an injury sustained during the race as noted in the Stewards’ Report. Where a horse otherwise died post-race or prior to a race (such as due to an injury sustained at the starting gates), the death was excluded. A horse fall was defined as a horse who fell during the race, including instances where a horse was brought down by another horse falling. The Victorian Racing Rules (LR93) require riders in jumps races to retire their horse in circumstances where the horse has fallen, is not in contention, is fatigued or distressed, or where continuing would increase the risk of a fall to the horse or rider [1]. Such occurrences were recorded as a failure to finish. A horse that was retired in a flat race was also recorded as having failed to finish. Where a horse was not retired, in contravention of rule LR93, this was recorded as a failure to retire. A horse contact occurred where the Stewards’ Reports noted that a horse had come into physical contact with another horse during the race.

Total injuries included data separately extracted for trauma (including lacerations, abrasions and epistaxis from trauma), lameness and exercise-induced pulmonary haemorrhage (EIPH), as described in the Stewards’ Reports. Where a horse was noted as having more than one injury, for example, multiple lacerations on a single leg or lacerations on multiple legs, these were counted as a single trauma. All injuries noted in the Stewards’ Report were included, regardless of severity. Where the Stewards’ Reports noted that a horse had a slower than normal recovery time or a horse was required to undergo a veterinary examination and obtain veterinary clearance that it was fit to race before the horse was permitted to start another race, these were counted as a slow recovery time and as veterinary clearance being required, respectively.

A dislodged rider was counted for each instance a rider fell from a horse during a race, regardless of whether the rider fall was associated with a horse fall. Whip breaches were those that incurred a reprimand or fine for failing to comply with the relevant Australian Racing Rules (AR132) or Victorian Racing Rules (LR41A), as noted in the Stewards’ Reports or JRP Reports.

This information was entered into an Excel spreadsheet. The number of horses starting each race type (starts) was tabulated. Data validation included checking a sample of data entries for accuracy against the Stewards’ Reports, checking for and correcting any illogical values, and verifying the formula used for tabulated data. The number of jumps race meets held each year and at each venue were cross-checked against publicly available information from the Australian Racing Fact Book and RV. The difference between the 77 jumps races reported to be held in 2021/2022 in the Australian Racing Fact Book 2022 [7] and the 73 races in 2022 in our dataset from the Stewards’ Reports reflects the financial year versus calendar year datasets, respectively. Where there was a discrepancy in the published Victorian Jumps Racing Programs and the Race Results/Stewards’ Reports, the Stewards’ Reports were used.

Data on horses withdrawn from the race prior to the start and post-race fatalities and injuries were excluded unless explicitly noted in the Stewards’ Reports as being attributable to the race. Training and trial fatalities and injuries were not consistently published and were therefore not considered. At the time of writing, Stewards’ Reports were not publicly available for three race meets in 2023, and JRP Reports were not publicly available for three meets in August 2022 and ten meets in 2023.

Only data from flat races held at race meets in which jumps races were also held were included in this study to mitigate the potential effects of seasonal impacts, such as ground and weather conditions, on race outcomes and to obtain similar sample sizes for both flat races and jumps races. Accordingly, data for flat races at race meets in the 2022 and 2023 racing seasons that held only flat races and no jumps races were not included. However, data for starts and fatalities in all flat starts for the 2021/22 and 2022/23 Victorian racing seasons were obtained from Racing Victoria’s 2022 and 2023 Annual Reports for context [25,26]. Seasonal data for starts and jockey rides were obtained from the Australian Racing Fact Book 2022 [7].

### 2.2. Data Analysis

Data were exported into SPSS (IBM SPSS Statistics v28; IBM Corporation, Armonk, NY, USA) to facilitate statistical analysis. Data were tabulated for each race type and incidence rates were calculated for fatalities, falls, failure to finish and total injuries on the basis of the number of horse starts per race type expressed as a rate per 1000 starts. Hurdle and steeplechase races were analysed separately, as prior studies reported statistically significant differences in the rates of horse fatalities, falls and injuries between these race types [14,27]. Incidence rates for jumps races overall were calculated by tabulating the data for hurdle and steeplechase races.

Given the data were categorical, contingency tables and chi-squared tests were used to assess the association between each outcome variable with the race type (flat, hurdle or steeplechase), month (March to August) and venue (Terang, Warrnambool, Hamilton, Pakenham, Casterton, Lakeside, Sale, Coleraine or Ballarat). Associations were considered statistically significant if the *p*-value was <0.05.

Outcome variables with significant associations were investigated to determine if these variables were more likely to be observed in each race type. Given that outcome variables were binary, multivariable binary logistic regression models were used. Variables that had greater than one outcome present in a single race were recategorized as having a single outcome status denoted as present (“1”) versus absent (“0”). Associations were considered statistically significant in the model if the *p*-value was <0.05. The results are reported as odds ratios (ORs) and 95% confidence intervals (CIs).

## 3. Results

### 3.1. Descriptive Statistics 

Data from 38 race meets comprising 307 races and 2816 horse starts were analysed. These races comprised 157 flats races (1597 starts) and 150 jumps races (1219 starts). Jumps races comprised 102 hurdle races (834 starts) and 48 steeplechase races (385 starts). The 38 race meets were held at nine regional tracks (Terang, Warrnambool, Hamilton, Pakenham, Casterton, Lakeside, Sale, Coleraine and Ballarat). Of the thirty-eight race meets, twelve were held at Warrnambool, six at Casterton, four at Lakeside, Pakenham and Hamilton, and two each at Ballarat, Coleraine, Sale and Terang. The six race meets at Pakenham and Ballarat held only jumps races and no flat races. The distribution of categorical variables is described in Table 1.

### 3.2. Statistical Analysis

Tabulation of the data showed fatalities, falls and total injuries to be more common in the jumps races compared with flat races (Table 1).

There were two fatalities each in hurdle and steeplechase races and no fatalities in flat races. Falls were more common in jumps races (*n* = 36, 3.0%), with no falls in flat races. Horses were pulled up (failed to finish) 119 times (9.8%) in jumps races compared with 4 times (*n* = 0.3%) in flat races. However, horse contacts occurred at a comparable rate in flat races (*n* = 80, 5.0%) and jumps races (*n* = 73, 6.0%). Overall injuries and each injury type (lame, trauma, EIPH, slow recovery time and veterinary clearance required) occurred more frequently in jumps races (*n* = 84, 6.9%) compared with flat races (*n* = 30, 1.9%).

Whip breaches were also more frequent in both hurdle (*n* = 15, 1.8%) and steeplechase race starts (*n* = 10, 2.6%) compared with flat race starts (*n* = 12, 0.8%). There were 10 (0.8%) instances of jockeys failing to retire a horse in jumps races. The frequency of dislodged riders in jumps races (*n* = 50, 4.1%) was also higher than in flat races (*n* = 2, 0.1%).

The rates of horse fatalities, falls, failure to finish and total injuries per 1000 horse starts were all higher in jumps races than in flat races (Table 2). The fatality rate in hurdles was 2.4 per 1000 starts and 5.2 per 1000 starts in steeplechase races. No fatalities occurred in flat races. The fall rate was 29.5 per 1000 starts in jumps races overall compared with no falls in flat races, while the rate of horses that failed to finish was 97.6 per 1000 starts in jumps races compared with 2.5 per 1000 starts in flat races. The rate of total injuries in jumps races was 68.9 per 1000 starts compared with 18.8 per 1000 starts in flat races.

Chi-squared tests were used to assess the association of each outcome variable with the race type, month and venue. Associations with the race type are presented in Table 3. Significant (*p* < 0.05) associations with the race type were found with falls, failure to finish, a dislodged rider, total injuries, trauma, veterinary clearance required and lameness. No significant associations with race type were observed with fatalities, whip breach, EIPH, slow recovery and horse contacts. Significant associations with venue were found with falls (*p* = 0.005), failure to finish (*p* = 0.012), a dislodged rider (*p* = 0.009) and slow recovery time (*p* = 0.002). Significant associations with the month were found with failure to finish (*p* = 0.046) and slow recovery time (*p* = 0.015).

Falls, failure to finish, a dislodged rider, total injuries, trauma, veterinary clearance required, and lameness were investigated using multivariable binary logistic regression models to determine if these outcomes were more likely to be observed in each race type (Table 4). The month, year and venue were evaluated as potential confounders of the associations with race type. Outcome variables were not associated with the month or year; however, there was evidence of association by venue. Venue-adjusted odds ratios are presented in Table 4.

In hurdle and steeplechase races, respectively, there were increased odds of total injuries (OR 4.0, 95% CI 2.1–7.3 and OR 5.2, 95% CI 2.5–11), trauma (OR 4.8, 95% CI 1.7–13.3 and OR 4.1, 95% CI 1.2–13.4), veterinary clearance required (OR 5.4, 95% CI 2.8–10.2 and OR 7.2, 95% CI 3.3–15.6) and lameness (OR 2.5, 95% CI 1.2–5.2 and OR 5.1, 95% CI 2.3–11.5), compared with flat races. A horse failing to finish was 44.7 times (*p* ≤ 0.001) and 68.7 times (*p* ≤ 0.001) more likely in hurdle and steeplechase races, respectively, compared with flat races. The odds of a dislodged rider were 20 times (*p* ≤ 0.001) more likely in hurdle races and 34.6 times (*p* ≤ 0.001) more likely in steeplechase races compared with flat races. However, the odds of horse falls were not significantly different for hurdle or steeplechase races compared with flat races.

## 4. Discussion

Overall, horse fatalities, falls and injuries occurred at higher rates in hurdle and steeplechase races compared with flat races in the 2022 and 2023 Thoroughbred horse jumps racing seasons in Victoria, consistent with prior studies in Australia [14,15,28]. This study reflected a reduction in the rates of horse fatalities, falls and injuries in jumps races compared with prior studies [14,15,28]. However, the rate of horse falls remained consistent, and the fatality rate in hurdle races was higher than that reported between 2012 to 2014 [14]. Fatalities, falls and injuries directly impact the welfare of horses and indirectly, the safety of riders. This study highlights the need for more comprehensive investigation into current risk factors for fatalities, falls and injuries for horses participating in jumps racing in order to develop tailored mitigation measures to improve the safety and welfare of horses and riders.

### 4.1. Fatalities

There were two horse fatalities in hurdle races and two in steeplechase races in Victoria during the study period, indicating an overall fatality rate in jumps races of 3.3 per 1000 starts, down from 5.1 per 1000 starts previously reported in Australia between 2012 and 2014 [14] and 8.3 per 1000 starts reported between 1989 and 2004 [15]. The fatality rate in this study period is at the lower end of fatality rates reported to be between 3.4 and 14.3 per 1000 starts collated from studies in Australia, the UK and the USA [13].

Consistent with horse fatality rates previously reported in Australia, the fatality rate in the study period was higher in steeplechase races than in hurdle races (5.2 and 2.4 per 1000 starts, respectively). Between 2012 and 2014, the fatality rates for steeplechase and hurdle races were 14 and 0.75 per 1000 starts, respectively [14]. Similarly, between 1989 and 2004, the odds of fatality in steeplechase starts were 2.5 times higher than hurdle starts [27] with a fatality rate of 6.3 per 1000 hurdle starts [15]. Industry measures were recommended to improve horse and rider safety targeted at steeplechase races in particular [14], and the fatality rate in steeplechase races in the study period reflects an improvement in the previously reported fatality rates in Australia. However, the results also reflect a higher fatality rate in hurdle races compared with the 0.75 per 1000 starts last reported for the 2012 to 2014 race seasons [14].

As there were no fatalities in flat races during the study period, the rate of horse fatalities in jumps races in the study period was effectively 3.3 times more than in flat races. The published rate of horse fatalities in the RV Annual Report for all flat races and jumps races in the 2022/23 financial year was 0.05% for flat races and 0.5% for jumps races [26]. Based on that financial year data, the rate of horse fatalities in jumps races was 10 times higher than in flat races in Victoria in 2022/23. The results from both the study period and from RV’s 2022/23 financial year data reflect an improvement compared with the 18.9 times higher rate previously reported for jumps races between 1989 and 2004 [27]. The improvement in the overall rate of horse fatalities in jumps races compared with flat races in Victoria, particularly in steeplechase races, supports claims that changes in racing rules and other measures implemented by the industry may have contributed to improved safety outcomes for horses. However, the rate of horse fatalities in jumps races remains higher than in flat races during the study period.

To support RV’s Equine Welfare Strategic Plan [29] and the development of supporting key performance indicators (KPIs) to reduce the rate of race day fatalities in jumps racing towards 0%, in line with targets for flat races [26], current risk factors for race day fatalities in jumps racing should be investigated. Measures such as the practical application of recent investigations into pre-race screening for musculoskeletal injuries and bone microdamage could be investigated [30,31,32,33]. Appropriate benchmarking of horse welfare may be facilitated by utilizing the available welfare assessment protocols [34].

### 4.2. Falls and Failure to Finish

Overall, the rate of horse falls in jumps races during this study was 29.5 per 1000 starts (*n* = 36, 3%), including two instances where a horse was brought down by another horse. The rates of horse falls in hurdle races (24 per 1000 starts) and steeplechase races (41.6 per 1000 starts) in the study period remain comparable with the rate of falls in hurdle and steeplechase races most recently reported in Australia in the 2012 to 2014 race seasons (29 and 40 per 1000 starts, respectively) [14].

For the 2012 to 2014 race seasons in Australia, these rates of horse falls were thought to indicate improvements from the implementation of changes in racing rules for hurdles and steeplechase, particularly the ability for the rider to withdraw the horse during the race (failure to finish) if fatigued and out of contention [14]. During that study period, the new rule was invoked in approximately 10% of starts. In New Zealand, a decrease in the rate of horse falls by 30% was observed in the eight seasons following the introduction of a similar rule in the 2011/12 season, with the number of horses who were pulled up during a race increasing linearly at a rate of ten horses per season [13]. This supported a prior finding in the UK between 1990 and 2010, in which the risk of a horse fall was reduced by 86% for every horse who was pulled up during the steeplechase race [35]. During this study period, this rule was also invoked for approximately 10% (*n* = 119) of jumps starts. While the rule does not apply to flat races, for flat races during this study, horses were retired from the race in 0.3% (*n* = 4) of starts. Jumps races reflected an increased risk of a horse being retired (failure to finish) from the race compared with flat races, being 44 times (OR 44.7, 95% CI 14.6–137.0) more likely in hurdle races and 68 times (OR 68.7, 95% CI 20.5–230.1) more likely in steeplechase races. While acknowledging that the rule does not apply in flat races, the results suggest that fatigue and injury leading to horses being retired from a race remain relevant factors for horses competing in jumps racing. Risk factors giving rise to horse fatigue and injury, such as the extent of training and experience and the number and timing of races entered [35,36], should be further investigated and mitigated where possible.

Despite the use of the rule to pull up horses who are fatigued and out of contention, the largely unchanged rate of horse falls since the 2012 to 2014 race seasons indicates that the rule may not be utilized as often as indicated or that other risk factors relevant to horse falls have not been adequately addressed by racing reforms to date. Risk factors associated with horse falls have been identified in prior studies in Australia, New Zealand and the UK. These include a higher rate of horse falls during the middle and end of the race [13], a longer race distance [13,37], an increasing number of horses in the race [37], the last jump of the race [13], the age and experience of the horse and age of jockeys [13,17,36], time since prior races [36], and whip use [16].

Amendments to the race distance, number of starters, removing the last jump, and the training and qualification requirements have been adopted in Victoria [1,14]. Similarly, the prior change to limit jumps racing to the cooler months, now run between March and August, potentially accounts for why falls were not associated with the month in which the race occurred in this study. Falls were, however, associated with the race venue (*p* = 0.005). A single race meeting at Ballarat accounted for seven of the 36 falls (including one of the two deaths in the hurdle races) in 2022 and a further two falls in 2023. There were nine falls at Warrnambool (where a death in steeplechase occurred), five at Pakenham, three at Lakeside, two at Sale and four at Hamilton (where the other deaths in hurdle and steeplechase occurred). While race distance has been found to be a risk factor over 4000 m in New Zealand, during this study period, the falls at Ballarat, Warrnambool and Pakenham (where most falls occurred) were during races held over distances from 3200 m to 5500 m, with only two races over more than 4000 m. Given the limited dataset, track-specific associations were not investigated; however, as risk factors have been associated with individual race tracks [35], a more comprehensive investigation of venue-specific risk factors could be carried out to identify and reduce the risk of falls.

In the UK, the risk of horse falls in hurdle and steeplechase races was associated with whip use and race progress [16]. Horses being whipped and progressing through the race were found to be at greater than seven times the risk of falling, compared to horses who were not being whipped and had lost their position or had no change in position [16]. A greater chance of falling was associated with two and three strikes of the whip [16]. Australian Racing Rule 132 requires that a rider must not use his or her whip in an excessive, unnecessary or improper manner, including when the rider’s horse is out of contention, showing no response, clearly winning or has no reasonable prospect of improving [4]. During this study period, there were approximately twice the instances of whip breach in jumps races compared with flat races, with 25 instances of whip breach in jumps racing (1.8% (*n* = 15) and 2.6%, (*n* = 10) of starts in hurdle and steeplechase races, respectively) compared with 12 instances (0.8%) in flat races. This study did not, however, find a significant association between whip breach and race type, and we did not investigate an association between permitted whip use and falls. The higher instances of whip breach in jumps racing, particularly in steeplechase races, warrants further investigation, given the association of horse falls with whip use in the prior UK study [16] and the increased risk of dislodged riders in jumps races.

Dislodged riders in jumps races in this study period represented 4.1% (*n* = 50) of starts. This is higher than the 1.6% of starts previously reported for the 2012 to 2014 race seasons [14] and comparable with 5.3% of rides between 2002 and 2006 [38]. In this study period, the risk of falls to riders was 20 times higher in hurdle races (OR 20, 95% CI 4.5–89.2) and 34.6 times higher in steeplechase races (OR 34.6, 95% CI 7.4–162) than in flat races. In prior studies, 50% of dislodged riders were associated with horse falls in jumps races in Australia [38] and 60% in New Zealand [39]. The risk of horse falls associated with whip use and risks associated with dislodged riders in jumps racing highlights an opportunity to further investigate an association between whip use, horse falls and dislodged riders, to underpin any modifications recommended to promote horse and rider safety.

Between 35 and 80% of horse fatalities have been reported to be associated with horse falls based on reported rates collated from studies in Australia, the UK and the USA [13]. While the two horse fatalities in hurdle races in this study period were not reported to be associated with falls, based on the Stewards’ Reports and JRP Reports, the horse fatalities in the steeplechase races were the result of serious race injuries sustained after falls at the last and penultimate obstacles. That is, fifty percent of horse fatalities during the study period were associated with horse falls. While RV stated that the introduction of One Fit hurdles resulted in a reduction in the 2021 fall rate by 66% on the 10-year average [10], horse falls in jumps races in the study period remain comparable to that in Victoria in the 2012 to 2014 race seasons, suggesting that there is further opportunity to investigate and implement ongoing improvements in both hurdles and steeplechase races.

### 4.3. Injuries

During the study period, injuries occurred at a rate of 68.9 per 1000 starts in jumps races compared with 18.8 per 1000 starts in flat races. This study found an increased risk of horse injuries overall in both hurdle and steeplechase races (OR 4.0, 95% CI 2.1–7.3 and OR 5.2, 95% CI 2.5–11, respectively) compared with flat races. Injuries requiring veterinary clearance were 5.4 times (OR 5.4, 95% CI 2.8–10.2) and 7.2 times (OR 7.2, 95% CI 3.3–15.6) more likely in hurdle and steeplechase races, respectively, compared with flat races. The odds of trauma were approximately 4 times more likely in hurdle and steeplechase races (OR 4.8, 95% CI 1.7–13.3 and OR 4.1, 95% CI 1.2–13.4, respectively) and the risk of lameness increased by 2.5 times in hurdle races (OR 2.5, 95% CI 1.2–5.2) and 5.1 times in steeplechase races (OR 5.1, 95% CI 2.3–11.5) compared with flat races. The risk of EIPH alone was not significantly different between jumps and flat races. According to the Stewards’ Reports, the fatalities in jumps races during the study period comprised horses euthanized or found dead in association with “serious racing injuries” and foreleg suspensory ligament injury. This suggests a rate of fatal musculoskeletal injuries (MSIs) in this study period of 3.3 per 1000 starts in jumps races overall (5.2 and 2.4 per 1000 starts in hurdle and steeplechase races, respectively).

Prior studies in Australia have reported the rates of catastrophic limb and cranial and vertebral injury in jumps races in Victoria [15] and fatal MSIs in flat races in NSW and the ACT [24]. In Victoria between 1989 and 2004, catastrophic limb injury caused 71.6% of racing fatalities across flat and jumps races and 69% of fatalities in jumps races [15]. Between 1988 and 1995, the risk of MSIs resulting in euthanasia or failure to return to racing for at least 6 months on city tracks in Victoria was 6.3 per 1000 starts in hurdle races and 14.3 per 1000 starts in steeplechase, compared with 0.6 per 1000 in flat races [28].

In flat races in NSW and the ACT between 2009 and 2014, the proportion of horse fatalities associated with MSIs was approximately 88% (0.52 of 0.59 per 1000 starts), which was reported to align with previous estimates of between 74.4 and 95% of fatalities [24]. In New Zealand, the incidence rate of all MSIs was 0.7 per 1000 starts and 0.4 per 1000 starts for fatal MSIs [40], lower than the pooled incidence of 1.17 per 1000 starts in a recent meta-analysis of flat races [41].

These studies reflect the continuing high risk of catastrophic limb injury or fatal MSI in both jumps racing and flat racing. While the studies to date have predominantly focused on catastrophic or fatal MSIs, injuries not directly resulting in death also pose a welfare concern for horses participating in racing. The results of this study indicate that horse injuries overall occur more commonly in jumps races compared with flat races. The results also suggest that the nature of the injuries in hurdle races and steeplechase races are potentially more severe, with increased odds of requiring veterinary clearance before returning to jumps racing.

RV has taken proactive steps to focus on physical welfare and reduce fatalities and career-ending injuries through its Equine Welfare Strategic Plan [29]. Baseline welfare targets should be developed by RV to be applied consistently across both jumps races and flat races, with research focused on current risk factors and tailored mitigation measures introduced to reduce the odds of injuries (and associated fatalities) in horses participating in jumps races to at least those in flat races. Innovative education and engagement strategies could also be considered as part of providing a broader education to industry stakeholders for the implementation of evidence-based horse- and rider-level training and measures that facilitate horse safety and welfare outcomes [42].

## 5. Limitations and Future Directions

This study was based on a retrospective dataset; therefore, the researchers had no influence on the completeness or accuracy of the information recorded. Stewards’ Reports may reflect potentially subjective or incomplete assessments with varying levels of detail and variation in the terminology used.

The dataset was limited to two jumps racing seasons and so caution must be exercised in interpreting and generalizing the findings. Risk factors such as weather conditions may have changed across years. A study of horse fatalities over 15 years found that the number of horse fatalities varied from year to year [15]. Analysis of data collected over a longer time period could more accurately ascertain the overall risk rate of fatalities, falls and injuries in jumps racing compared with flat racing in Victoria to determine any material change in risk since the prior study periods between 2012 and 2014 [14] and 1989 and 2004 [15]. In addition, some confidence intervals in the final multivariable logistic regression model were wide. Ongoing monitoring, data collection and analysis are warranted, particularly to understand risk factors for deaths, since only four events were observed during the study period.

Due to the parameters set at the commencement of this study, we did not include fatalities and injuries in horses related to training or trials, horses withdrawn prior to the race (scratched), or incidents which led to post-race euthanasia or retirement that were not reported in the Stewards’ Reports. Therefore, the findings likely underestimated the total number of fatalities or injuries associated with jumps racing. While the Australian Racing Rules and Victorian Racing Rules require the reporting of all deaths, including those occurring during training and trials, this information is not consistently made publicly available. Future studies could assess the incidence of fatalities that occur post-race, in training, trials and horse retirements associated with jumps racing in collaboration with data provided by RV and Racing Australia, to help provide a more accurate assessment.

Similarly, while the overall number of race day fatalities is now reported annually in the 2023 Racing Victoria Annual Report “Safety Record” [26], publicly available data continue to be recorded on a race-by-race basis through Stewards’ Reports and are not otherwise aggregated [26]. Aggregating the data would help to identify trends and comprehensively assess potential risk factors at a track level in future studies using a planned selection of different venues to represent different track types, where observation can occur during the same period. This would facilitate the development of targeted track-level mitigation strategies.

Although the focus of this study was on fatalities, falls and injuries impacting horses, the results showed that jumps races represented a significantly increased risk of riders being dislodged compared with flat races. Future studies could build upon the studies of dislodged riders in 2011 and 2014 [24,36] to assess the current rates and severity of injuries to riders participating in jumps races, including the relative economic impact of these workplace injuries on the sustainability of the sport and consequently, the financial capacity to improve horse welfare [6].

To support the continual implementation of best practice welfare measures concurrently with the pursuit of higher racing performance, knowledge of the current risk factors for horse fatalities, falls and injuries and tailored mitigation measures to improve horse and rider safety should be incorporated into the development of evidence-based training programs for industry participants, veterinarians, and veterinary and animal science students [42].

## 6. Conclusions

Horse fatalities, falls and injuries occurred at higher rates in jumps races compared with flat races in the 2022 and 2023 Thoroughbred horse jumps racing seasons in Victoria. Horse falls occurred at similar rates to those previously reported. This was the case even for horses who were withdrawn due to fatigue or out of contention (failed to finish) in approximately 10% of jump starts. Horse injuries occurred at higher rates in hurdle races and steeplechase races with trauma being at least 4 times more likely. The risk of lameness was 2.5 and 5 times more likely in hurdle and steeplechase races, respectively, and the requirement for veterinary clearance, at least 5 times more likely than in flat races.

These findings suggest that further investigation of current risk factors for horse fatalities, falls and injuries for horses participating in jumps racing is warranted. This would allow for tailored mitigation measures towards improving the safety and welfare of horses and riders. A consistent, transparent and publicly available reporting framework for horse fatalities, falls and injuries should be considered to support the implementation of the Equine Welfare Strategic Plan, including by facilitating the monitoring of long-term trends and determining the efficacy of measures introduced to improve horse and rider safety.

Given the known heightened and inherent risks of jumps racing for both horses and riders, ongoing investment in further studies and measures to improve safety would assist the Victorian Thoroughbred horse jumps racing industry to secure its social license into the future. In the context of horse and rider safety, without such ongoing investment, the sustainability of jumps racing, which now represents only 1.8% of Thoroughbred horse races in Victoria, may be at risk.

## Figures and Tables

**Table 1:** Frequency of outcome variables in Thoroughbred horse flat, hurdle and steeplechase races in Victoria during the 2022 and 2023 jumps racing seasons (*n* = 307) and percent of starts (*n* = 2816).

	Flat		Hurdle		Steeplechase		Jumps ^2^	
No. of Races	157		102		48		150	
No. of Starts ^1^	1597		834		385		1219	
	Number	Percent of Starts	Number	Percent of Starts	Number	Percent of Starts	Number	Percent of Starts
Fatalities	0	0.0	2	0.2	2	0.5	4	0.3
Falls	0	0.0	20	2.4	16	4.2	36	3.0
Failed to Finish	4	0.3	71	8.5	48	12.5	119	9.8
Horse Contacts	80	5.0	54	6.5	19	4.9	73	6.0
Dislodged Rider	2	0.1	28	3.4	22	5.7	50	4.1
Whip Breach	12	0.8	15	1.8	10	2.6	25	2.1
Total Injuries	30	1.9	52	6.2	32	8.3	84	6.9
Lame	15	0.9	21	2.5	20	5.2	41	3.4
Trauma	8	0.5	18	2.2	8	2.1	26	2.1
EIPH ^3^	7	0.4	13	1.6	4	1.0	17	1.4
Slow Recovery Time	23	1.4	15	1.8	4	1.0	19	1.6
Veterinary Clearance Required	25	1.6	53	6.4	34	8.8	87	7.1
Failed to Retire	0.0	0.0	6	0.7	4	1.0	10	0.8

^1^ Starts were calculated as the total number of horses starting in each race type. ^2^ Jumps were calculated by tabulating the data for hurdle and steeplechase races. ^3^ Exercise-induced pulmonary haemorrhage.

**Table 2:** Horse fatality rate, fall rate, failed to finish rate and total injury rate (calculated per 1000 starts) in Victoria during the 2022 and 2023 jumps racing seasons for flat, hurdle and steeplechase races (*n* = 2816 starts).

	Flat	Hurdle	Steeplechase	Jumps ^2^
No. of Races	157	102	48	150
Starts ^1^	1597	834	385	1219
Fatality Rate	0.0	2.4	5.2	3.3
Fall Rate	0.0	24	41.6	29.5
Failed to Finish Rate	2.5	85.1	124.7	97.6
Total Injury Rate	18.8	62.4	83.1	68.9

^1^ Starts were calculated as the total number of horses starting in each race type. ^2^ Jumps were calculated by tabulating the data for hurdle and steeplechase races.

**Table 3:** Crosstabulation and Pearson chi-squared results for race type for horse fatalities, falls, failed to finish, whip breach, dislodged rider, total injuries, trauma, EIPH, veterinary clearance required, slow recovery time, horse contacts and lame, in Victoria during the 2022 and 2023 jumps racing seasons for flat, hurdle and steeplechase races (*n* = 307 races). Statistically significant (*p* < 0.05) associations are indicated in bold and with an *. Crosstabulation of single outcome status is denoted as present (“1”) versus absent (“0”).

	Race Type	0	1	Pearson’s Chi-Square	*p*-Value	Likelihood Ratio	Degrees of Freedom
Fatalities	Flat	157	0	5.5	0.07	6.4	2
	Hurdle	100	2				
	Steeplechase	46	2				
Falls *	**Flat**	**157**	**0**	**41.5**	**<0.001**	**49.9**	**2**
	**Hurdle**	**86**	**16**				
	**Steeplechase**	**34**	**14**				
**Failed to** Finish *	**Flat**	**153**	**4**	**97.3**	**<0.001**	**112.5**	**2**
	**Hurdle**	**52**	**50**				
	**Steeplechase**	**20**	**28**				
Whip Breach	Flat	147	10	3.9	0.145	3.8	2
	Hurdle	90	12				
	Steeplechase	41	7				
Dislodged Rider *	**Flat**	**155**	**2**	**43.8**	**<0.001**	**49.9**	**2**
	**Hurdle**	**79**	**23**				
	**Steeplechase**	**32**	**16**				
**Total** Injuries *	**Flat**	**131**	**26**	**23.7**	**<0.001**	**24.1**	**2**
	**Hurdle**	**62**	**40**				
	**Steeplechase**	**26**	**22**				
Trauma *	**Flat**	**151**	**6**	**13.1**	**0.001**	**13.8**	**2**
	**Hurdle**	**85**	**17**				
	**Steeplechase**	**41**	**7**				
EIPH ^1^	Flat	150	7	4.8	0.090	4.8	1
	Hurdle	90	12				
	Steeplechase	44	4				
**Veterinary Clearance** Required *	**Flat**	**134**	**23**	**30.6**	**<0.001**	**31.3**	**2**
	Hurdle	61	41				
	Steeplechase	25	23				
Slow Recovery	Flat	134	23	1.4	0.502	1.5	2
	Hurdle	87	15				
	Steeplechase	44	4				
Horse Contacts	Flat	123	34	0.6	0.740	0.6	2
	Hurdle	80	22				
	Steeplechase	40	8				
Lame *	**Flat**	**143**	**14**	**17.3**	**<0.001**	**16.4**	**2**
	**Hurdle**	**82**	**20**				
	**Steeplechase**	**32**	**16**				

^1^ Exercise-induced pulmonary haemorrhage.

**Table 4:** Crosstabulation and summary of statistically significant (*p* < 0.05) venue-adjusted associations from multivariable logistic regression analysis of horse failed to finish, total injuries, trauma, dislodged rider, veterinary clearance required and lame, in Victoria during the 2022 and 2023 jumps racing seasons for hurdle and steeplechase races compared with flat races (*n* = 307 races). The table sets out the estimated coefficient (B), standard error (S.E.), Wald chi-squared test (Wald), degrees of freedom (Df), *p*-value (Sig.), odds ratio and 95% confidence interval (CI)’s lower and upper values for odds ratio.

								95% CI	
	Race Type	B	S.E.	Wald	Df	Sig.	Odds Ratio	Lower	Upper
Failed to Finish	Hurdle	3.8	0.6	44.2	1	<0.001	44.7	14.6	137.0
	Steeplechase	4.2	0.6	46.9	1	<0.001	68.7	20.5	230.1
Total Injuries	Hurdle	1.4	0.3	18.9	1	<0.001	4.0	2.1	7.3
	Steeplechase	1.6	0.4	18.7	1	<0.001	5.2	2.5	11.0
Trauma	Hurdle	1.6	0.5	9.2	1	0.002	4.8	1.7	13.3
	Steeplechase	1.4	0.6	5.4	1	0.021	4.1	1.2	13.4
Dislodged Rider	Hurdle	3.0	0.8	15.4	1	<0.001	20.0	4.5	89.2
	Steeplechase	3.5	0.8	20.3	1	<0.001	34.6	7.4	162
Veterinary Clearance Required	Hurdle	1.7	0.3	26.6	1	<0.001	5.4	2.8	10.2
	Steeplechase	2.0	0.4	25.3	1	<0.001	7.2	3.3	15.6
Lame	Hurdle	0.9	0.4	5.9	1	0.015	2.5	1.2	5.2
	Steeplechase	1.6	0.4	15.4	1	<0.001	5.1	2.3	11.5

## Data Availability

Data are available from the Results and Stewards’ Reports area of the Racing.com, last accessed on 9 December 2023, website and the Jumps Review Panel Race Reports (JRP Reports) available on the RV website (https://www.racingvictoria.com.au/the-sport/racing/jumps/jumps-review-panel, last accessed on 9 December 2023).
